# Application of Bézier Curves for Calculating Likelihood Ratios for Plasma Amyloid-β Biomarkers for Alzheimer's Disease

**DOI:** 10.3389/fnagi.2018.00276

**Published:** 2018-10-02

**Authors:** Walter Fierz

**Affiliations:** Labormedizinische Zentrum Dr Risch, Bern, Switzerland

**Keywords:** Alzheimer's disease, Bézier curves, biomarkers, likelihood ratios, ROC curves

## Abstract

**Introduction:** Alzheimer's disease, being the most frequent cause of dementia in elderly people, still is difficult to diagnose and to predict its occurrence. The clinical application of biomarkers for diagnosis of Alzheimer's disease has been restricted so far to the analysis of proteins in the cerebrospinal fluid like amyloid β_1−42_ and p-tau. However, in a recently published nature letter it has been shown that the high-performance measurement of amyloid-β in plasma alone could provide a method well suited for a broad clinical application. The study uses ROC analysis to evaluate the clinical significance of the method but it does not provide likelihood ratios (LR) of the measured results.

**Methods:** In this article, a newly developed method is used to calculate LRs for any measurement result of a study by approximation of the ROC curves using Bézier curves. Such LRs provide an estimation of the clinical significance of any particular test result by applying Bayes' theorem: Pretest odds for disease multiplied by the LR of the test result give the posttest odds.

**Results:** The application of the Bézier curve approximation to the data of the plasma amyloid-β study is demonstrated. To generalize the calculation of LRs for all test results, a relation between the test results and the points on the Bézier curve with their LRs is established.

**Discussion:** The application of Bézier curves in ROC analysis allows calculating LRs for all individual test results when measuring amyloid-β biomarkers for Alzheimer's disease.

## Background

The clinical application of biomarkers for diagnosis of Alzheimer's disease (Prince et al., 2013; Scheltens et al., 2016) is mainly based on the analysis of amyloid β_1−42_ and p-tau in the cerebrospinal fluid (Ewers et al., 2015; Skillbäck et al., 2015). However, to avoid the necessity of gaining cerebrospinal fluid it would be desirable to have diagnostic tests based on peripheral blood. Biomarkers in the peripheral blood have so far only been found with a proteomic approach (Doecke et al., 2012; Lista et al., 2013). Now, in a recent publication it has been shown that the high-performance measurement of amyloid-β in plasma alone could provide a method for clinical application (Nakamura et al., 2018). However, the study using ROC analysis for evaluation of the clinical significance of the method does not provide likelihood ratios (LR) of the measured results. LRs are defined by the frequency of a particular test result in the diseased vs. non-diseased population and are used to estimate the clinical significance of the test results. LRs serve to calculate the posttest odds for Alzheimer's disease by multiplying the pretest odds with the calculated LR of the test according to Bayes' theorem (Van Der Helm and Hische, 1979).

When a cut-off is defined to distinguish positive from negative results of a test, the ROC analysis provides the sensitivity (Se) and specificity (Sp) of such qualitative results. LRs of positive and negative results are LR^+^ = Se/(1-Sp) and LR^−^ = (1-Se)/Sp. However, these LRs only are an average over all positive or negative results. When looking at a particular quantitative test result, it would be desirable to know the LR of that particular result. On the ROC curve such a LR is equal to the slope of the tangent to the ROC curve at the point that corresponds to the test result (Choi, 1998). LRs of test results higher than the optimal cut-off range between 1 and ∞, whereas LRs of negative test results range between 1 and 1/∞. Remarkably, the plasma amyloid-β study (Nakamura et al., 2018) provides all raw data of the ROC curves, so that such LRs can be calculated based on these data. Here, a newly developed method is used to calculate LRs for any quantitative test results by approximation of the ROC curves using Bézier curves (Fierz, 2018).

## Methods

The data used from the plasma amyloid-β study (Nakamura et al., 2018) concern the ^11^C-labeled Pittsburgh compound-B - positron-emission tomography (PiB-PET) data set, since the primary aim of the study was to assess the performance of plasma-Aβ biomarkers for determining an individual's status of Aβ deposition, using PiB-PET as the standard of truth. The results of the study were externally validated using an independent data set derived from the Australian Imaging, Biomarker and Lifestyle Study of Ageing (AIBL) cohort (Ellis et al., 2009).

In order to calculate the LR of a specific plasma amyloid-β test result, the tangent to the ROC curve at the point corresponding to the point on the ROC curve has to be determined. For this and for calculating the slope of the tangent, Bézier curves can be used, as recently described (Fierz, 2018) and is demonstrated here. The mathematical basis for Bézier curves are the Bernstein polynomials (Casselman, 2008). Bernstein polynomials of degree n are defined by

$$\begin{array}{l} B_{i,n}\left(t\right)=\left(\begin{array}{c} n\\ i \end{array}\right)t^{i}{\left(1-t\right)}^{n-i},\text{with t ranging from }0\;\mathrm{to}\;1. \end{array}$$

**Figure 1:**
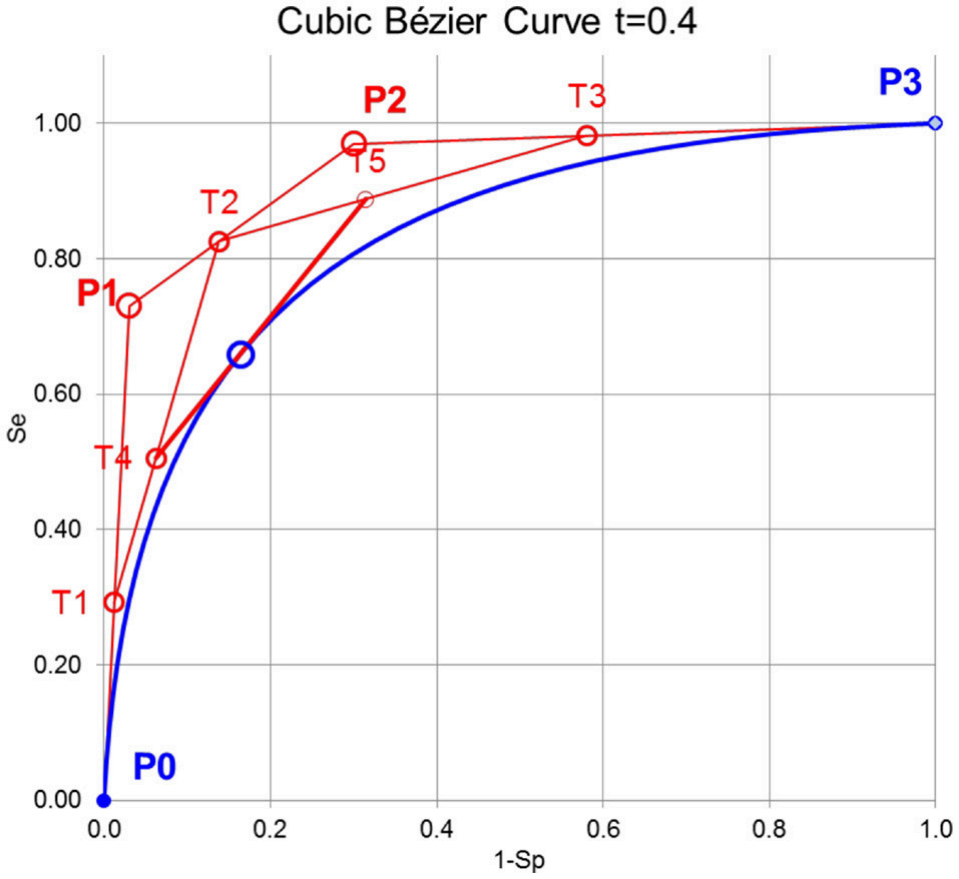
Principle of constructing cubic Bézier curves. First, the lines between the control points P_0_, P_1_, P_2_, and P_3_ are divided by the variable t of the Bernstein polynomial leading to T_1_, T_2_, and T_3_. Second, the lines between T1, T2, and T3 are again divided by t leading to T4 and T5. Third, the line between T4, and T5 is again divided by t leading to B(t) on the Bézier curve. The line between T4, and T5 is the tangent to B(t).

For the purpose here, cubic Bézier curves are used, which are defined by 4 control points P_0_, P_1_, P_2_, and P_3_ and a variable t that define the tangent to a specific point on the curve as described in Figure 1. The cubic Bernstein polynomial is

$$\begin{array}{l} \text{B}\left(\text{t}\right)={\left(1-\text{t}\right)}^{3}{\text{P}}_{0}+3\text{ t}{\left(1-\text{t}\right)}^{2}{\text{P}}_{1}+\;3\;{\text{t}}^{2}\left(1-\text{t}\right)\;{\text{P}}_{2}+{\text{t}}^{3}{\text{P}}_{3} \end{array}$$

The variable t of the Bernstein polynomials has to be introduced and is defined here by *t* = (1-Sp+Se)/2 of the empirical ROC points. With the de Casteljau algorithm (Casselman, 2008) it is possible to construct a Bézier curve or to find a particular point on the Bézier curve (Figure 1).

## Results

**Figure 2:**
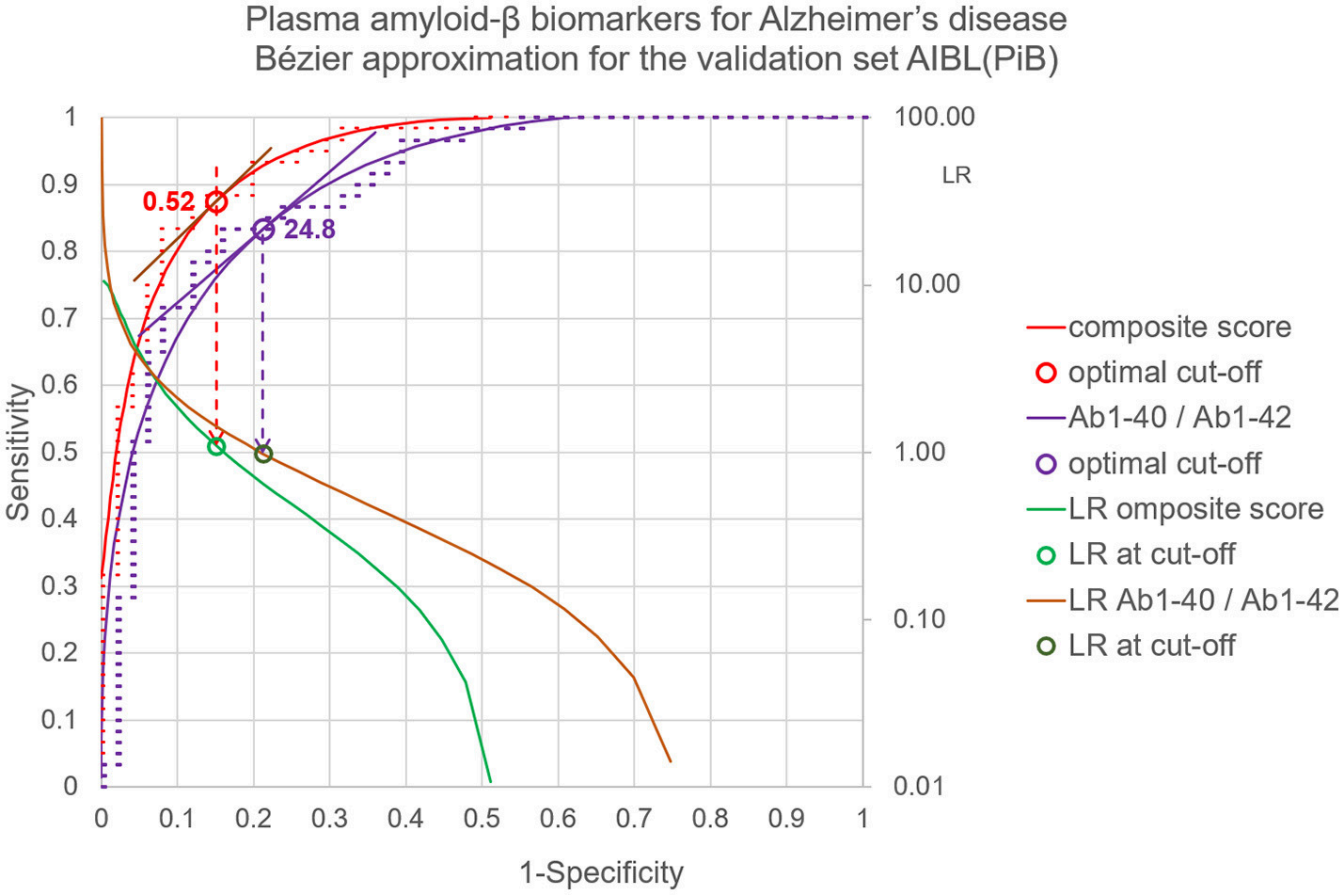
Bézier curve approximation to the data of the plasma amyloid-β study. LRs of individual points of the Bézier curve are calculated as the slopes of the tangents to the points: LR = (T5_y_-T4_y_)/(T5x–T4x) (see Figure 1). At the optimal cut-off point at a composite score of 0.5 and a Aβ_1−40_/Aβ_1−42_ ratio of 25 the LR = 1.

The application of the Bézier curve approximation to the data of the plasma amyloid-β study is demonstrated in Figure 2. The x and y parameters for P_0_, P_1_, P_2_, and P_3_ are calculated according to the method of (Fierz, 2018) and are displayed in Table 1 for the composite score and Aβ_1−40_ / Aβ_1−42_ ratio in the AIBL(PiB) validation data set of the plasma amyloid-β study.

**Table 1:** Bézier curve parameters.

**Validation: AIBL(PiB)**	**Composite score**		**Ab_1−40_/Ab_1−42_ Ratio**	
	**x**	**y**	**x**	**y**
P0	0.000	0.312	0.000	0.014
P1	0.039	0.727	−0.007	0.755
P2	0.067	0.995	0.192	1.067
P3	0.511	1.000	0.961	1.000

To generalize the calculation of LRs for all test results, a relation between the test results and the points on the Bézier curve with their LRs had to be found. The best approximation was found by introducing a parameter λ = 1/(1+LR) and using a cubic regression between test results x and λ resulting in:

λ = ax^3^ + bx^2^ + cx + d with coefficients given in Table 2.

**Table 2:** Coefficients of the cubic regression between test results x and λ.

**x**	**a**	**b**	**c**	**d**	**R2**
Aβ_1−40_/Aβ_1−42_	0.00015189	−0.010320125	0.154661878	0.69895132	0.9984
Composite score	−1.009861	2.267549089	−2.448933781	1.25118718	0.9942

**Figure 3:**
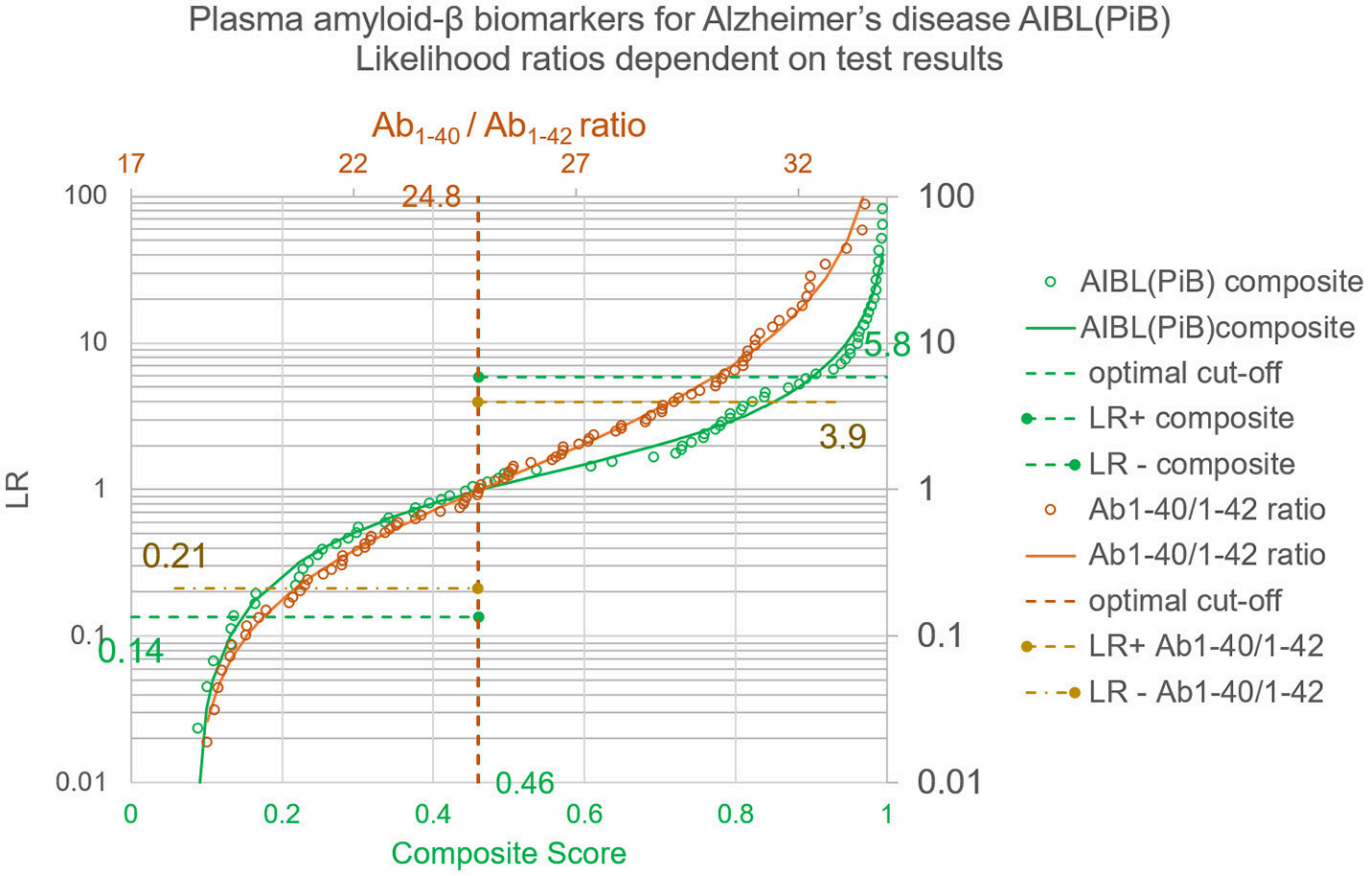
Likelihood ratios for test results of the plasma amyloid-β study. Whereas the LR^+^ and LR^−^ provide an average over all positive or negative results, the LRs dependent on quantitative test results and calculated with the Bézier curve parameters of Table 1 give a value for each individual test result. The circles correspond to the LRs of the empirical ROC points, the lines show the generalized relation between test results and LRs as calculated with λ = 1/(1+LR) and the coefficients shown in Table 2.

The thereby calculated LR = (1–λ)/λ are depicted in Figure 3.

In the following, two examples are calculated using Bayes' theorem (Van Der Helm and Hische, 1979):

Pretest odds for disease multiplied by the LR of the test result give the posttest odds.

First, taking the prevalence of ~7% in people over 60 years in Western Europe or North America (Prince et al., 2013) as a pretest probability Table 3 shows the posttest odds and probabilities for various Aβ_1−40_/Aβ_1−42_ ratios.

**Table 3:** Posttest odds and probabilities for various Aβ_1−40_/Aβ_1−42_ ratios based on pretest odds.

**Pretest**		**Ab_1−40_/Ab_1−42_**			**Posttest**	
**Prevalence**	**Odds**	**Ratio**	**λ**	**LR**	**Odds**	**Probability%**
7%	7:93 = 0.075	20	0.879	0.14	0.010	1
		23	0.645	0.55	0.041	4
		25	0.489	1.05	0.079	7
		27	0.341	1.93	0.145	13
		30	0.152	5.60	0.421	30
**Probability**	**Odds**	**Aβ_1−40_/Aβ_1−42_**	λ	**LR**	**Odds**	**Probability%**
50%	1:1 = 1	20	0.879	0.14	0.137	12
		23	0.645	0.55	0.551	36
		25	0.489	1.05	1.047	51
		27	0.341	1.93	1.932	66
		30	0.152	5.60	5.596	85

*Second, assuming pretest odds of 1:1, Table 3 shows the posttest odds and probabilities for various Aβ_1−40_/Aβ_1−42_ ratios*.

## Conclusions

A distribution-free method, based on Bézier curves, to calculate likelihood ratios for quantitative laboratory test results in medical diagnosis (Fierz, 2018) is applied here for Alzheimer's diagnosis based on the results of the plasma amyloid-β study (Nakamura et al., 2018). The advantage of the method is that it is generally applicable independently and without knowledge of the test parameter distribution in the population. The crucial benefit of this procedure is that Bézier curves are constructed by tangents to the ROC curve, whose slopes immediately provide the likelihood ratios of a specific point on the curve. The merit of using likelihood ratios in addition to or even instead of quantities like mg/L or nmol/L as test results lies in the comparability of different test methods and different test suppliers, which is an unsolved problem of standardization in laboratory medicine.

In conclusion, the application of Bézier curves in ROC analysis (Fierz, 2018) allows to calculate LRs for all individual test results when measuring amyloid-β biomarkers for Alzheimer's disease. LRs allow estimating the posttest odds for Alzheimer's disease by multiplying the pretest odds with the calculated LR. Such LRs provide an estimation of the clinical significance of any particular test result by applying Bayes' theorem (Van Der Helm and Hische, 1979):

Pretest odds for disease multiplied by the LR of the test result give the posttest odds.

## Data availability statement

All relevant data are within the paper or in referenced source.

## Author contributions

The author confirms being the sole contributor of this work and has approved it for publication.

### Conflict of interest statement

The author declares that the research was conducted in the absence of any commercial or financial relationships that could be construed as a potential conflict of interest.

## Abbreviations

ROC: Receiver Operating Characteristics
LR: Likelihood Ratio
Se: sensitivity
Sp: specificity
PiB-PET: Pittsburgh compound-B - positron-emission tomography
AIBL: Australian Imaging, Biomarker and Lifestyle Study of Ageing.
